# Parylene Based Memristive Devices with Multilevel Resistive Switching for Neuromorphic Applications

**DOI:** 10.1038/s41598-019-47263-9

**Published:** 2019-07-25

**Authors:** Anton A. Minnekhanov, Andrey V. Emelyanov, Dmitry A. Lapkin, Kristina E. Nikiruy, Boris S. Shvetsov, Alexander A. Nesmelov, Vladimir V. Rylkov, Vyacheslav A. Demin, Victor V. Erokhin

**Affiliations:** 1 National Research Centre “Kurchatov Institute”, 123182 Moscow, Russia; 2 Moscow Institute of Physics and Technology, 141700 Dolgoprudny, Moscow Region Russia; 3 Lomonosov Moscow State University, 119991 Moscow, Russia; 4 Kotel’nikov Institute of Radio Engineering and Electronics RAS, 141190 Fryazino, Moscow Region Russia; 5 CNR-IMEM (National Research Council, Institute of Materials for Electronics and Magnetism), 43124 Parma, Italy; 6 Present Address: Deutsches Elektronen-Synchrotron DESY, 22607 Hamburg, Germany

**Keywords:** Materials science, Neuroscience

## Abstract

In this paper, the resistive switching and neuromorphic behaviour of memristive devices based on parylene, a polymer both low-cost and safe for the human body, is comprehensively studied. The Metal/Parylene/ITO sandwich structures were prepared by means of the standard gas phase surface polymerization method with different top active metal electrodes (Ag, Al, Cu or Ti of ~500 nm thickness). These organic memristive devices exhibit excellent performance: low switching voltage (down to 1 V), large OFF/ON resistance ratio (up to 10^4^), retention (≥10^4^ s) and high multilevel resistance switching (at least 16 stable resistive states in the case of Cu electrodes). We have experimentally shown that parylene-based memristive elements can be trained by a biologically inspired spike-timing-dependent plasticity (STDP) mechanism. The obtained results have been used to implement a simple neuromorphic network model of classical conditioning. The described advantages allow considering parylene-based organic memristors as prospective devices for hardware realization of spiking artificial neuron networks capable of supervised and unsupervised learning and suitable for biomedical applications.

## Introduction

Memristive devices are of great research interest nowadays owing to a number of their attractive properties such as low energy consumption and voltage operation, high write/read rate, multilevel resistive switching (RS) and ability to store intermediate states as well as manufacturability due to their simple two-terminal structure, and low cost of fabrication^1–4^. Among various applications of memristive devices, the most promising are resistive random-access memory (RRAM) fabrication^1^, computing in memory^2^, and neuromorphic computing^3–10^. The multilevel character of RS, or the possibility of “analog” resistance variation in the window between the low- and high-resistance states (*R*_on_, *R*_off_), is one of the most important properties of memristors for emulating synapses (key elements of biological neural networks that couple neurons with variable weight functions) in the development of neuromorphic computing systems like the human brain (in terms of pattern and speech recognition, learning ability, and other cognitive tasks)^2,3^.

It is also important that there are organic memristive structures that could be fabricated on flexible biocompatible substrates, used for neuroprosthetics and so-called “wearable” electronic devices^5,11,12^. Stable operation of flexible memristive devices was shown at rather small bending radii (down to 9 mm) and longitudinal twisting up to 30 degrees^13,14^. Moreover, resistance in this case can be tuned in the window between *R*_on_ and *R*_off_ according to the rules similar to those in biological neural networks^5^, in particular, the so-called “spike-timing-dependent plasticity” (STDP) first implemented for inorganic memristive structures^15^.

The memristive properties were found in structures based on inorganic oxides (TiO_x_, HfO_x_, SiO_x_, TaO_x_, etc.)^16–22^, organic (polyaniline, polythiophene)^23–25^ and nanocomposite^26^ materials. Most memristive devices operate through the electromigration of oxygen vacancies in oxides and formation (rupture) of conductive filaments (valence change memristors) or metal bridge growth (destruction) by means of cation motion in a dielectric matrix (electrochemical metallization or ECM memristors)^1^. In the last case an electrochemically active metal, for example Ag, is used as one of the electrodes of the memristive structure of metal-insulator-metal (MIM) type. Under applied positive voltage, Ag^+^ cations can migrate to the cathode, where they are reduced and form metal bridges^1,5,27–34^. This mechanism can also be realized with Cu^34–36^ and Al^37–39^ active electrodes. Moreover, the conductive filaments can consist of carbon formed by local pyrolysis of organic material^40,41^.

One of the most promising memristive structures for “wearable” applications at the present moment are connected to MIM structures based on polymeric layers of parylene (poly-para-xylylene, or PPX) due to the simple and cheap production of this polymer, its transparency and the possibility of the fabrication on flexible substrates^39,42^. Moreover, parylene is an FDA-approved material and could be used in biomedical applications since it is completely safe for the human body, which cannot be said about most of the other organic materials^14,39,43^. Parylene-based materials have found the widest applications in electronics and electrical engineering, and, especially, in radio-electronic equipment production. Parylene is used as protective coating and an insulating layer in integrated circuits and thin-film transistors, microelectromechanical systems, lasers, waveguides and photodiodes^44–46^.

Currently, parylene-based memristive structures have shown a fairly wide window of RS (*R*_off_/*R*_on_~10^4^) and reasonable retention^39,42^. However, the possibility of their “analog” resistance variation, or plasticity (an analog of synaptic plasticity in biological systems), as well as their implementation in neuromorphic systems, has not been reported yet. Therefore, the main goals of this study are: 1) to develop a technology for fabrication of memristive structures based on parylene layers with multilevel resistance switching (high plasticity); 2) to study their memristive properties, as well as the STDP-like learning ability; 3) to demonstrate the ability of parylene-based memristors to implement synapse-like elements in spiking neuromorphic networks with memristive STDP learning.

## Materials and Methods

We have studied memristive elements of Metal/Parylene/ITO structure (further M/PPX/ITO structure). The parylene layers (~100 nm) were deposited on a commercially purchased ITO coated glass substrate (bottom electrode) by the gas phase surface polymerization method using SCS Labcoater PDS 2010 vacuum deposition system. PDS 2010 transforms Parylene dimer (2,2-para-cyclophane or its derivatives) to a gaseous monomer; the material polymerizes onto the substrate upon deposition at room temperature. There is no intermediate liquid phase or separate cure cycle. At the vacuum levels employed, all sides of the substrate were uniformly impinged on by the gaseous monomer, resulting in a truly conformal coating.

The top metal electrodes were Ag, Al, Cu or Ti layers (~500 nm) obtained by thermal evaporation or ion-beam sputtering through a shadow mask. The sizes of the top electrodes were 0.2 × 0.5 mm^2^, and about 150 devices (per one substrate) were fabricated for each kind of electrode. The metals listed above were selected due to their widespread use in electronic engineering including the manufacture of memristive devices.

The structural investigations were carried out with the transmission electron microscope (TEM) Titan 80–300 (FEI, USA) in TEM and STEM modes. An energy dispersive X-RAY analyser (EDX, USA) was used to reveal the chemical composition. The cross-sectional preparation of the memristive samples was done by the focused ion beam (FIB) method on Helios 600i.

Memristive characteristics (I-V curves, *R*_off_/*R*_on_ ratio, plasticity, retention time, endurance) of the M/PPX/ITO structures were studied using a Cascade Microtech PM5 analytical probe station; the voltage pulses were supplied by a National Instruments PXIe-4140 source measure unit, programmed in LabView. All experiments were performed at room temperature.

## Results and Discussion

### Structure

**Figure 1:**
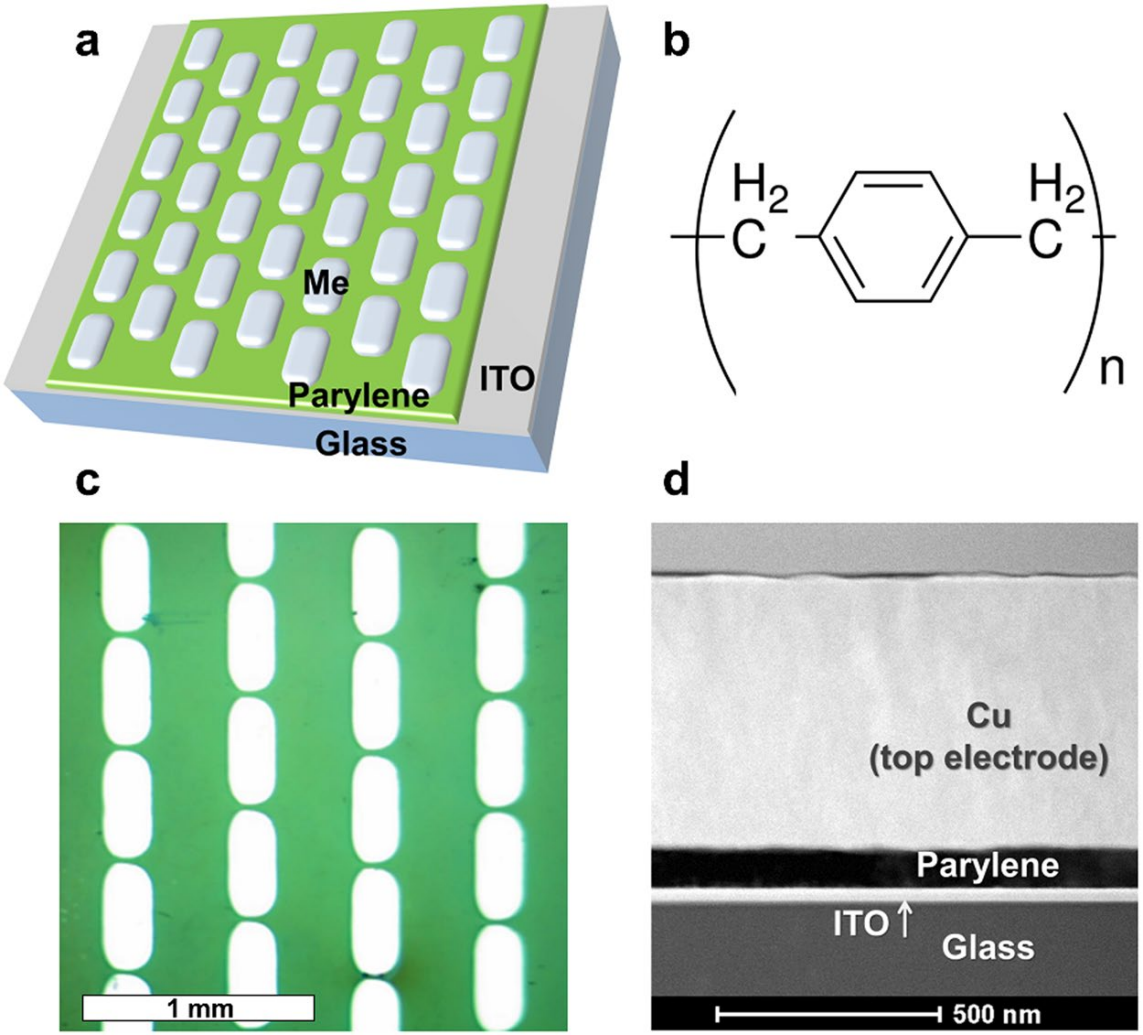
(**a**) Scheme of the M/PPX/ITO memristive structure. (**b**) Parylene N repeat unit. (**c**) Microscopic image of Cu/PPX/ITO memristive elements, constituting the memristive structure (only a part of the sample is shown). (**d**) Layers of the Cu/PPX/ITO memristive element, a TEM image.

The schematic view of the fabricated thin-film memristive structure is shown in Fig. 1(a). In all the electrical experiments described in this work, voltage is applied to the top electrode (Ag, Cu, Al or Ti), while the bottom electrode (ITO) is grounded. Figure 1(b) represents the chemical structure unit of parylene (active layer). Figure 1(c,d) show the top view microscopic image and cross sectional TEM image of the Cu/PPX/ITO memristive structure, respectively.

### Memristive characteristics

**Figure 2:**
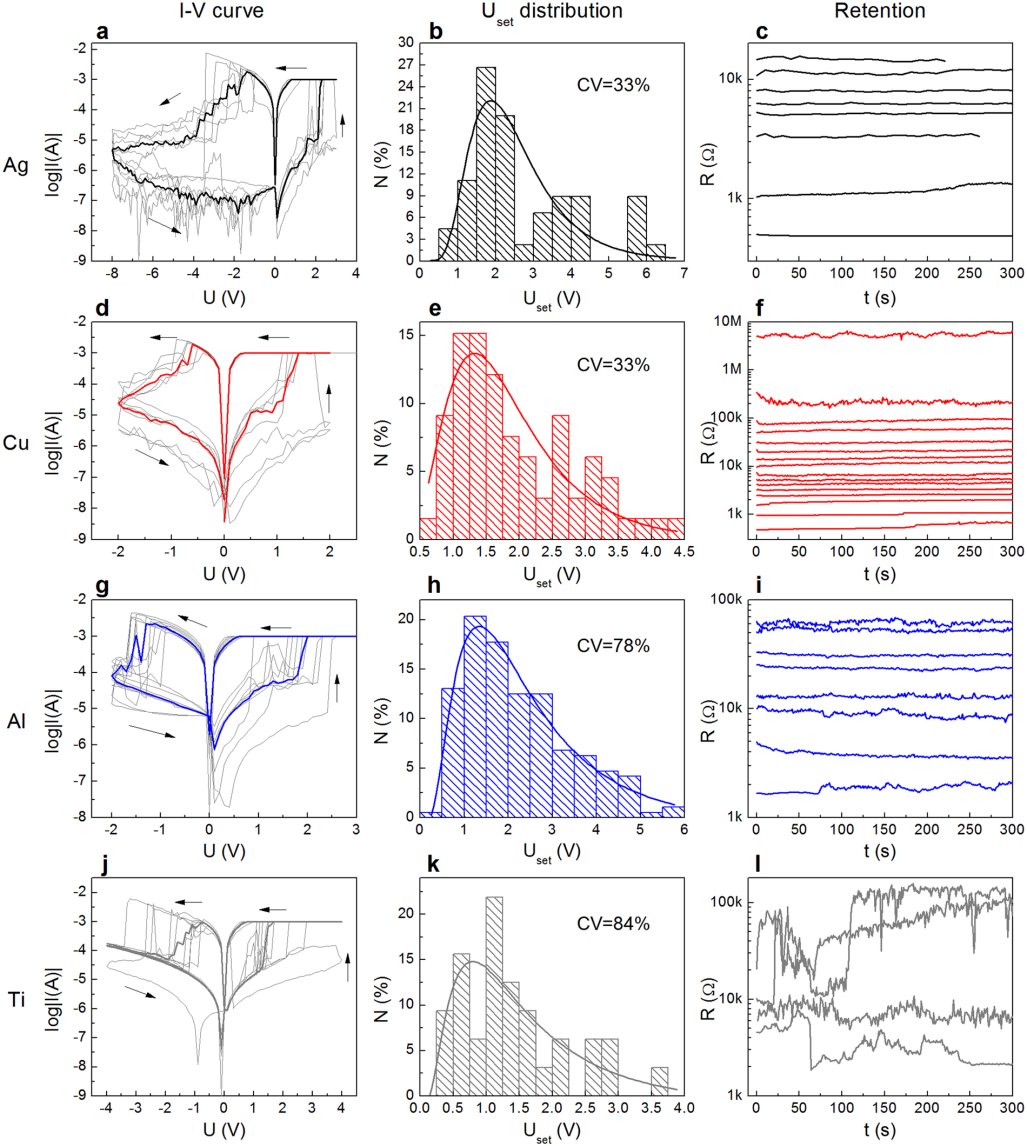
Memristive properties of (Ag, Cu, Al, Ti)/PPX/ITO structures: typical I-V curves (cycle-to-cycle, the median is highlighted in bold), *U*_set_ distributions (~100 cycles) and retention time (*U*_read_ = 0.1 V) characteristics.

The resistive switching characteristics of the samples could be observed from the typical cyclic I-V curves, shown in Fig. 2. Compliance currents of +1 and −100 mA were set to prevent overheating and subsequent breakdown of the structures. Each cycle was carried out by applying a voltage sweep that included a rise up to a positive voltage *U*_+_ sufficient to switch the M/PPX/ITO device to the low-resistance state (LRS) (0 → *U*_+_ → 0) and, after that, a fall down to a negative voltage *U*_−_ to switch it back to the high-resistance state (HRS) (0 → *U*_−_ → 0). The sweep rate was 2 V/s (with a step of 0.1 V). All the samples were initially in the HRS with resistances of ~10^5^, ~10^6^, ~10^5^ and ~10^5^ Ω for Ag, Cu, Al and Ti electrodes, respectively. The LRS was ~1 kΩ for all the samples.

As shown by the I-V curve in Fig. 2(a), the current through the Ag/PPX/ITO sample (further we will denote it as “Ag sample”, the other samples will be denoted similarly) suddenly increases to the compliance level at ~2 V. On the other hand, the RESET voltage was relatively high equalling ~−4 V. The device exhibits stable bipolar RS behaviour with *R*_off_/*R*_on_~30. The “cycle-to-cycle” reproducibility of the I-V characteristics of the Ag sample can be characterized by the SET voltage distribution (Fig. 2(b)), representing data from ~100 I-V measurements. The maximum of the log-normal fit of this distribution is ~2 V; its mean value and standard deviation are 2.1 and 0.7 V, respectively (coefficient of variation CV = 33%).

**Figure 3:**
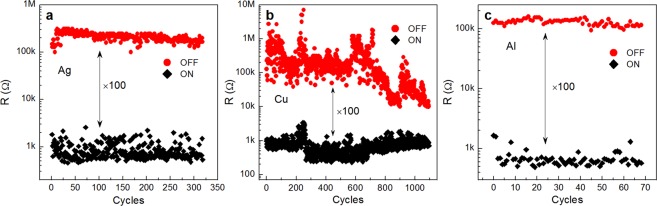
Endurance of (**a**) Ag/PPX/ITO, (**b**) Cu/PPX/ITO and (**c**) Al/PPX/ITO memristors. Black and red points represent low- and high-resistance states, respectively. Pulse time was 100 ms; U_set_ = −U_reset_ = 5 V for (**a**), U_set_ = −U_reset_ = 4 V for (**b**) and U_set_ = 5 V, U_reset_ = −8 V for (**c**).

Figure 2(c) demonstrates the plasticity of the Ag sample. For programming the required resistive state, a high precision (no worse than 0.5%) iterative algorithm^47^ was used, based on the application of voltage pulses with smoothly varying amplitude and duration of 100 ms (see Supplementary Note 1 for details). As one can see from the figure, at least 8 five-minute stable resistive states (which do not intersect over time) were clearly observed between 500 Ω and 15 kΩ, what makes it possible to write at least 3 bits of information into a single memory element (the minimal number of stable resistive states was estimated in a series of retention experiments). Such stability should be enough for most short-term memory applications, but these structures also show a long-time (more than 10^3^ s) retention of low- and middle-resistance states (~700 and ~5000 Ω, see Supplementary Fig. S1). The Ag samples also show acceptable endurance (Fig. 3(a)): more than 300 cycles of stable switching between LRS and HRS (1 and 100 kΩ, respectively). There are also prospects for increasing the number of stable resistive states since the *R*_off_*/R*_on_ value in the endurance experiment is higher than in the retention one. It should be noted that we did not observe any RS processes in Ag samples with the thickness of parylene layer ≥200 nm; they remained in the HRS with *R* = 10^7^–10^8^ Ω even when the voltage applied to the Ag electrode reached 10 V. It may indicate that the dielectric parylene coat is too thick in that case for the metal bridge to grow^1,39^.

I-V characteristics of Cu samples (Fig. 2(d)) show a definitely better cycle-to-cycle reproducibility than in the case of the Ag top electrode. The values of *U*_set_ are distributed (Fig. 2(e)) with the maximum of the log-normal fit at ~1.3 V. The mean *U*_set_ value of this distribution is 1.5 V, which is less than in the case of the Ag sample. The standard deviation of *U*_set_ is 0.5 V, so the CV equals 33%. It is worth noting that the CV could be tuned by reducing the size of the memristor top electrode or by introducing additional barrier layers (e.g. graphene^42^). On the other hand, a relatively high CV of *U*_set_ is preferable for hardware-intrinsic security primitives (e.g. physical unclonable functions^48^). We note that the *U*_set_ and *U*_reset_ parameters almost stabilize after several cycles, forming sharp-switching and symmetrical hysteretic curves. These facts are promising in terms of neuromorphic applications (because such symmetric pulses can be used for learning). Moreover, the Cu samples exhibit good plasticity; as one can see from the retention curves in Fig. 2(f), at least 16 stable resistive states (4 bits of information) can be programmed in memristors of this kind. It should be noted that the *R*_off_/*R*_on_ ratio reaches 10^4^ in this experiment. Despite the fact that Fig. 2 shows only short-time retention curves (5 min), the long-living states (up to 5 h) were also recorded (see Supplementary Fig. S2). The samples also demonstrate good endurance, but the gap between HRS and LRS tends to decrease slowly after ~600 cycles from the value of *R*_off_/*R*_on_ = 10^2^–10^3^ down to ~10 (see Fig. 3(b)). It may be due to the features of the measuring algorithm used: during the measurement the sample is constantly under an alternating voltage stress, which may cause its overheating and possible degradation. Going forward, we want to emphasize that in the STDP-like learning process, where dozens of RSs to a state with preset resistance take place (according to the accurate algorithm^47^), the Cu sample exhibits good performance showing more than 10^4^ precise switchings without failure, which is the best result among the samples investigated in this work.

Regarding Al samples, they have also demonstrated good cycle-to-cycle reproducibility but the *U*_set_ distribution is wider (CV = 78%) than in the case of the Cu electrode (CV = 33%). It was estimated from the log-normal fitting (Fig. 2(h)) that the mean value of *U*_set_ equals here 2.7 V with the standard deviation of 2.1 V. It is too wide for neuromorphic applications. Moreover, the Al samples have worse plasticity than the Cu ones: only 8 stable resistive states were detected (Fig. 2(i)). Their endurance is also poor: less than 100 cycles. In spite of these facts, these structures show reasonable short-time retention (see Fig. 2(i)).

The Ti samples, as shown in Fig. 2(j), do not demonstrate any of the desired features: their I-V curves are asymmetric and irreproducible, the plasticity is absent due to the poor retention, and the endurance is too noisy and insufficient (therefore it is not presented in Fig. 3).

General comparison of memristive characteristics of all the samples is shown in Table 1. Note that the Cu samples are superior in all parameters, especially in plasticity. This could be explained by taking into account the higher surface activation energy of Cu compared to Ag and Al, which leads to a smaller diffusion rate^34^. Thus, the lifetime of Cu bridges is longer and, hence, the plasticity of Cu samples is better. This result is also confirmed by a recent study by Lübben *et al*.^49^, where the filament stability and retention properties of ECM devices were found to be determined by the Gibbs free energy of formation of cations.

**Table 1:** Memristive Characteristics of M/PPX/ITO Samples.

Sample	max R_off_/R_on_	U_set_ (μ ± σ), V	Number of resistive states	Endurance, cycles
Ag/PPX/ITO	100	2.1 ± 0.7, CV = 33%	8	300
Cu/PPX/ITO	10 000	1.5 ± 0.5, CV = 33%	16	1000
Al/PPX/ITO	100	2.7 ± 2.1, CV = 78%	8	70
Ti/PPX/ITO	50	1.9 ± 1.6, CV = 84%	—	—

Note that although the question of the nature of RS in M/PPX/ITO structures is extremely interesting, its detailed analysis lies beyond the scope of this study. Here, based on the experimental data and on the related literature, we only make the following conclusion: the bipolar RS behaviour of the investigated samples most likely originates from the formation/destruction of metal bridges (filaments) in the dielectric parylene layer due to migration of metal cations (Ag^+^, Cu^+^/Cu^+2^, Al^+3^ or Ti^+4^) from the top electrode toward the bottom during the SET process under strong electric field^1,3,34,39,49^. In contrast, according to the molecular dynamics simulations performed by Wang *et al*.^34^, the conductive bridges can spontaneously break during the RESET switching as a result of atomic surface diffusion driven by the minimization of the system energy.

### Memristive spike-timing-dependent plasticity

**Figure 4:**
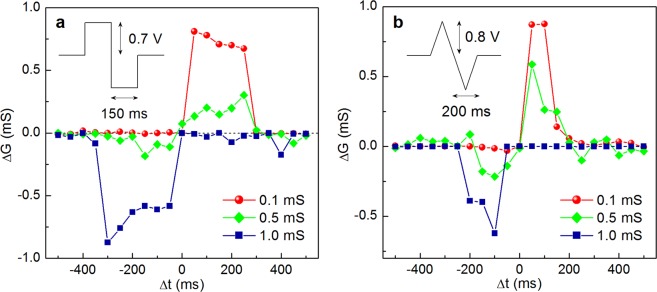
STDP window of Cu/PPX/ITO memristive structures (for various initial conductance values) obtained with heteropolar (**a**) bi-rectangular and (**b**) bi-triangular spike pulses shown in the figure insets. Post-synaptic spikes were applied after (before) pre-synaptic ones with a varying delay time Δ*t*. Every point of the curves is a median of 10 recorded experimental values.

For the STDP-like learning experiments we chose the Cu samples because they showed the best memristive characteristics among the investigated structures. The bottom electrode (ITO) of the Cu/PPX/ITO memristive structure was assigned for the pre-synaptic input and the top electrode (Cu) was considered as the post-synaptic one. We used identical voltage pulses as pre- and post-synaptic spikes of heteropolar bi-rectangular (inset in Fig. 4(a)) or bi-triangular (inset in Fig. 4(b)) shape. The amplitudes of bi-rectangular and bi-triangular spikes were chosen to be 0.7 V and 0.8 V, respectively, so the spike itself could not lead to a conductivity change in the structure. On the other hand, if two spikes are summed up, the voltage drop across the memristive device could be increased up to ±1.4 and ±1.6 V, which is within the switching range of the Cu sample. The pulse half-durations were 150 and 200 ms with discretization of 50 ms. Post-synaptic pulses were applied after (before) pre-synaptic pulses with varying delay time Δ*t* (ranged from −500 to 500 ms with a step of 50 ms).

Conductance was measured by the application of a testing voltage of +0.1 V within 50 ms before and after the sequence of pre- and post-synaptic pulses. Generally, the device conductance *G* is regarded as a synaptic weight, and then its change (Δ*G*) is equal to a synaptic weight change. More specifically, a weight change corresponds to Δ*G* = *G*_*f*_ − *G*_*i*_, where *G*_*f*_ and *G*_*i*_ are the final and the initial conductance values, respectively. Thus, the determined weight change dependence on the delay time (memristive STDP window) for two different initial states is shown in Fig. 4.

One can see from Fig. 4 that the experimental results obey the rule similar to STDP one, observed in biological systems^50^. Synaptic potentiation (Δ*G* > 0) was observed for Δ*t* > 0, and synaptic depression (Δ*G* < 0) was observed for Δ*t* < 0. Note that the result of STDP-like learning depends on the *G*_*i*_ value. If a memristor state is close to LRS, then its synaptic weight would likely depress rather than potentiate (as for the 1 mS state in Fig. 4), and vice versa (0.1 mS state in Fig. 4). On the other hand, when the memristor initially was in the intermediate state (0.5 mS), learning curves demonstrated both synaptic potentiation (up to 120% for Δ*t* > 0) and depression (down to –44% for Δ*t* <0). This “multiplicative” character of the memristive STDP curve can be explained by taking into account the finiteness of a conductance change in the studied memristors.

It should be noted that we tested a wide range of pulse amplitudes (0.5, 0.6, 0.7, 0.8 and 0.9 V) and half-durations (50, 100, 150, 200 and 250 ms) for several resistive states of the Cu sample. These results can be found in Supplementary Information (Supplementary Figs S3–S8).

### Neuromorphic application of STDP-like learning

**Figure 5:**
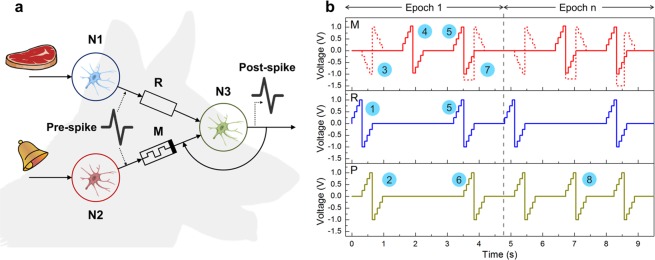
STDP-like learning memristive Pavlov’s dog implementation. (**a**) The electrical schematic diagram: N1 — the 1st pre-neuron, spiking after the “food”-related stimulus; N2 — the 2nd pre-neuron, spiking after the “bell” stimulus; N3 — the post-neuron, which spikes when the total input current exceeds the threshold; R — a resistor with a constant resistance value *R* = 2 kΩ; M — a memristive element, initially in the *R*_off_ = 20 kΩ resistive state. A post-spike is generated unconditionally after a spike comes from N1 and under the condition that the memristor current exceeds *I*_th_ after a spike comes from N2. (**b**) An example of the spike pattern applied to the inputs of the scheme: 1 — the initial pulse (1st Epoch) on the resistor (R) (unconditioned stimulus), resulting in post-spike (P) 2, which in turn comes to the memristor (M) as pulse 3 (dashed) in the inverted form; 4 — the pulse on the memristor, initially without post-neuron activity; 5 — simultaneous pulses on the resistor and the memristor, which result in post-spike 6 leading to the teaching pulse 7 (dashed); 8 — a post-spike as a result of the conditioned stimulus when the training is completed (Epoch *n*, where *n* is equal to or above the number of epochs needed for successful conditioning).

After successful experimental implementation of STDP-like learning for parylene-based memristive structures, we would like to take a step forward to demonstrate their utility in constructing simple neuromorphic networks. For this purpose, we have chosen the task of classical (also known as Pavlovian) conditioning^51,52^ and constructed a network (simulating Pavlov’s dog behaviour) consisting of 2 pre-synaptic neurons connected with a post-synaptic one (Fig. 5(a)). The first pre-synaptic neuron is connected to the post-synaptic one via a resistor *R* corresponding to an unconditioned stimulus (e.g. “food”) pathway. The second pre-synaptic neuron connection is represented by a memristive element (Cu/PPX/ITO) corresponding to an initially neutral stimulus (e.g. a “bell”) pathway. Each neuron was implemented in software: the pre-synaptic neurons were programmed to generate spikes of amplitude *U*_sp_ (the spike shape was similar to that in the inset of Fig. 4(b)), and the post-synaptic one was used as a threshold unit (generating spikes only in the case of the total input current exceeding the threshold current *I*_th_, which is chosen to be slightly less than the ratio *U*_sp_/*R*). The bottom electrode of the memristive element was connected to the output of the post-synaptic neuron. A similar electronic implementation of Pavlov’s dog has been presented before^53^, however there was used constant-signal learning without the use of any STDP-like rules. Another implementation was proposed in a network with pseudo-memcapacitive synapses with a Hebbian-like learning mechanism^54^.

The learning procedure was as follows: 1) introducing a signal only down the unconditioned stimulus pathway (by this step we check the correct post-synaptic neuron activity, i.e. the dog starts “salivating”, having been exposed to the sight (or smell) of “food”); 2) sending a signal only down the conditioned stimulus pathway (in this step we check whether an initially neutral stimulus becomes a conditioned one); 3) pairing the two stimuli (in this step the conditioning (learning) occurs). These three steps constituted one epoch of learning shown schematically in Fig. 5(b).

Resistance *R* = 2 kΩ (Fig. 5(a)) was chosen to be slightly greater than the *R*_on_ resistive state of the Cu/PPX/ITO memristive structure (~1 kΩ) in order to provide the possibility of successful training of the network. The spike amplitudes *U*_sp_ and durations *Δt*_sp_ were identical for all neurons and were selected experimentally according to the results of the I-V and STDP-like learning measurements (see previous sections). Every experiment started with the *R*_off_~20 kΩ state of the memristor (which was, in fact, an intermediate state, because resistance in the “true” HRS reaches 5 MΩ, see Fig. 2(f)). As is shown in Fig. 5(b), when the conditioned stimulus is paired with the unconditioned one (Fig. 5(b), spikes 5), the post-synaptic pulse (spike 6) from N3 (which starts being generated when the memristor current exceeds the current threshold *I*_th_) sums up with the pre-synaptic pulse from N2, leading to a resistivity change in the memristor (by the dashed spike 7). If the introduction of the “bell” signal alone resulted in the post-synaptic neuron spiking (spike 8), the conditioning occurred.

**Figure 6:**
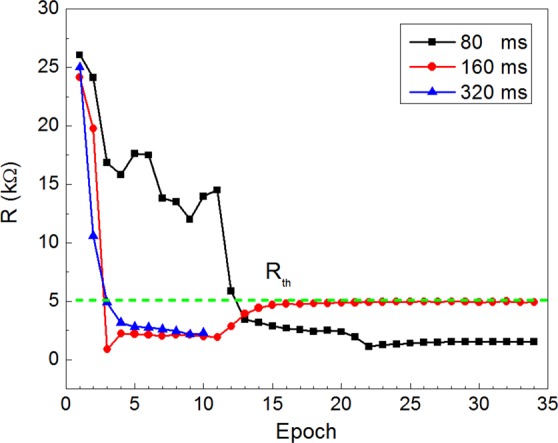
Electronic Pavlov’s dog implementation: memristor resistance depending on the number of learning epochs.

In our experiments the parameters were such that the threshold resistance value of the memristor that led to neuron spiking (i.e. the highest resistance value when *U*_sp_/*R*_th_ > *I*_th_) was *R*_th_~5 kΩ. We used spikes of heteropolar bi-triangular shape with the amplitude of 1 V and the half-duration of 80, 160 and 320 ms. It can be seen from Fig. 6 that the conditioning occurred in all the cases, but the number of epochs necessary for conditioning was different depending on the pulse durations. Namely, the shorter was the pulse duration used, the larger was the number of epochs necessary for successful conditioning. This may be due to the fact that the short duration of the electric field influence (80 ms) is not enough to form a continuous metal bridge. Since the effect of local heating can be neglected here (a relatively long time passes between the pulses — about 1 s), the total pulse time remains the only parameter determining the conditioning rate (assuming constant amplitude of learning pulses). The obtained results demonstrate the associative learning ability of neuromorphic systems with parylene-based memristive elements and provide a basis for the development of autonomous circuits capable of emulating cognitive functions.

## Conclusions

In conclusion, we have successfully demonstrated that parylene-based memristive devices are capable of learning (including learning using biologically inspired STDP-like rules). The Metal/Parylene/ITO structures were fabricated with Ag, Cu, Al and Ti top active electrodes. These organic memristive devices (except for those with Ti top electrodes) exhibit the advantages of low switching voltage (down to 1 V), high *R*_off_/*R*_on_ ratio (up to 10^4^), long retention time (≥10^4^ s) and multilevel resistance switching (at least 16 stable resistive states in the case of Cu electrodes). We have experimentally shown that parylene-based memristive elements can be trained by the spike learning mechanism. The model of classical conditioning (electronic “Pavlov’s dog”) was implemented as a simple neuromorphic circuit using Cu/Parylene/ITO memristors. The obtained results demonstrate the associative learning ability of neuromorphic systems with parylene-based devices, which is especially valuable given that parylene is a polymer that is FDA-approved and completely safe for the human body. Thus the development of memristive systems based on it provides prospects for hardware realization of artificial neural networks for wearable and biomedical applications.

### Public availability

A copy of the manuscript is freely available online as a pre-print at arxiv.org: https://arxiv.org/abs/1901.08667.

## Supplementary information

Parylene Based Memristive Devices with Multilevel Resistive Switching for Neuromorphic Applications

## Data Availability

The datasets obtained and analysed during the current study are available from the corresponding author on reasonable request.
