# Supplementary material for: Parylene Based Memristive Devices with Multilevel Resistive Switching for Neuromorphic Applications
Source: Sci Rep. 2019 Jul 25;9:10800. doi: 10.1038/s41598-019-47263-9 (PMC6658497; doi:10.1038/s41598-019-47263-9)
Supplement: Supplementary file 1 — Parylene Based Memristive Devices with Multilevel Resistive Switching for Neuromorphic Applications [file 41598_2019_47263_MOESM1_ESM.pdf]

Supplementary Information

Parylene Based Memristive Devices with Multilevel Resistive Switching for Neuromorphic Applications

Anton A. Minnekhanov[1], *Andrey V. Emelyanov*[1,2], *Dmitry A. Lapkin*[1,†], *Kristina E. Nikiruy*[1,2], *Boris S. Shvetsov*[3], *Alexander A. Nesmelov*[1], *Vladimir V. Rylkov*[1,4], *Vyacheslav A. Demin*[1,2], *Victor V. Erokhin*[1,5,*]

[1]National Research Centre "Kurchatov Institute", 123182 Moscow, Russia.

[2]Moscow Institute of Physics and Technology, 141700 Dolgoprudny, Moscow Region, Russia.

[3]Lomonosov Moscow State University, 119991 Moscow, Russia.

[4]Kotel'nikov Institute of Radio Engineering and Electronics RAS, 141190 Fryazino, Moscow Region, Russia.

[5]CNR-IMEM (National Research Council, Institute of Materials for Electronics and Magnetism), 43124 Parma, Italy

[†]Present address: Deutsches Elektronen-Synchrotron DESY, 22607 Hamburg, Germany.

*E-mail: victor.erokhin@fis.unipr.it

Supplementary Note 1

Details of the precise algorithm of memristor switching to a state with preset resistance

The algorithm of memristor switching to a state with preset resistance [1] that was used is based on the application of voltage pulses with smoothly varying amplitude and duration of 100 ms. The main input parameters of this algorithm are amplitude of the first pulse U_0, pulse amplitude change ΔU at the next step, preset memristor resistance R, accuracy $\Delta R/R$ of resistance setting, and duration τ of the learning pulses. The essence of the algorithm is as follows. Every ith iteration is divided into two stages: measuring and learning. At each stage, the resistance R_i is calculated by applying a reading pulse (+0.1 mV, 50 ms). At the learning stage, pulses with amplitudes U_i and duration 100 ms are applied, where the first learning pulse amplitude is always U_0 and the polarity of all learning pulses coincides with the sign of the $(R_i - R)$ difference. Then, if the signs of $(R - R_{i-1})$ and $(R - R_i)$ are the same, the amplitude of the ith learning pulse is changed to $U_i = U_{i-1} + \Delta U$; if the signs are different, the amplitude is $U_i = U_0$ and only the ith pulse polarity is changed. If amplitude U_i at the ith step exceeds the maximum permissible value, the amplitude is reset to U_0. The algorithm operation is terminated if the current structure resistance falls within the preset accuracy interval ($R - \Delta R < R_i < R + \Delta R$).

[1] Nikiruy, K. E. *et al.* A precise algorithm of memristor switching to a state with preset resistance. *Tech. Phys. Lett.* **44,** 416–419 (2018).

Long-time retention

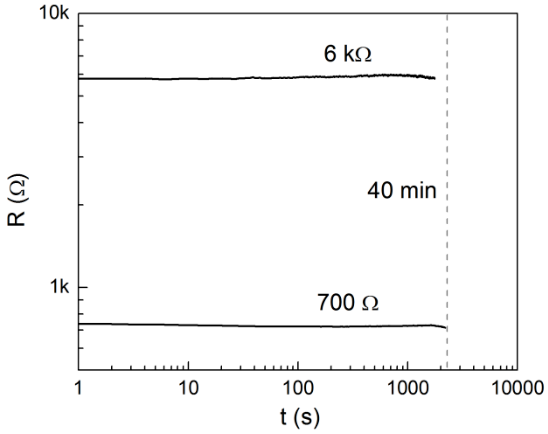

Figure S1. Long-time retention of Ag/PPX/ITO memristors.

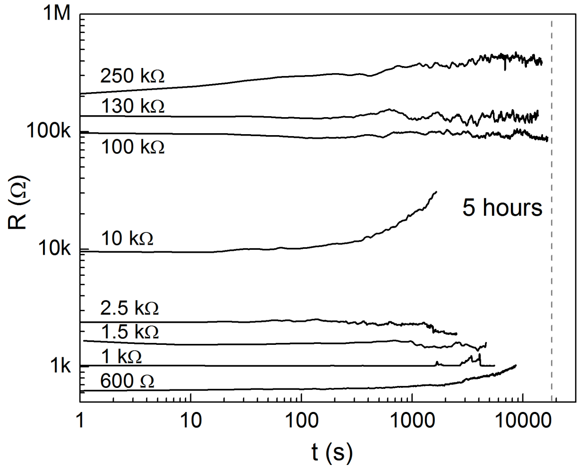

Figure S2. Long-time retention of Cu/PPX/ITO memristors.

Spike learning

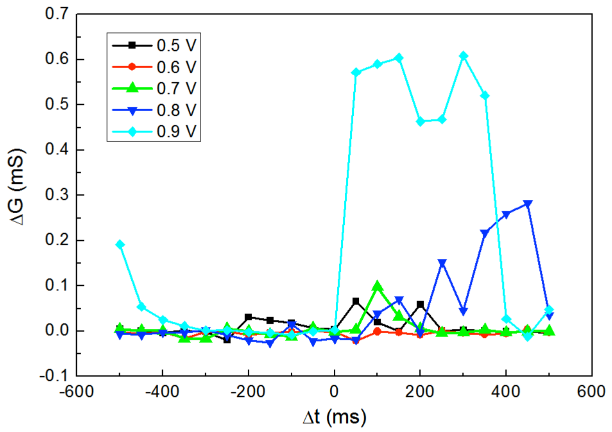

Figure S3. STDP window of Cu/PPX/ITO memristive structures, obtained with bi-rectangular pulses of various amplitudes. Post-synaptic pulses were applied after pre-synaptic ones with a varying delay time Δt. All the pulses were symmetric. Every point of the curves is a median of 10 recorded experimental values. Initial conductance was 0.1 mS, pulse half-durations were 200 ms.

As one can see, the best result is obtained when the pulses of 0.7 V are applied (the shape of the curve is smooth and it has one extremum).

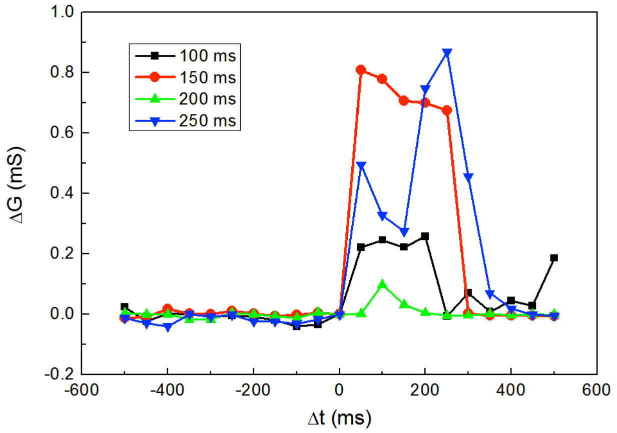

Figure S4. STDP window of Cu/PPX/ITO memristive structures, obtained with bi-rectangular pulses of various half-durations. Post-synaptic pulses were applied after pre-synaptic ones with a varying delay time Δt. All the pulses were symmetric. Every point of the curves is a median of 10 recorded experimental values. Initial conductance was 0.1 mS, pulse amplitudes were 0.7 V.

As one can see, the best result in this case is obtained when the pulses of 150 ms are applied.

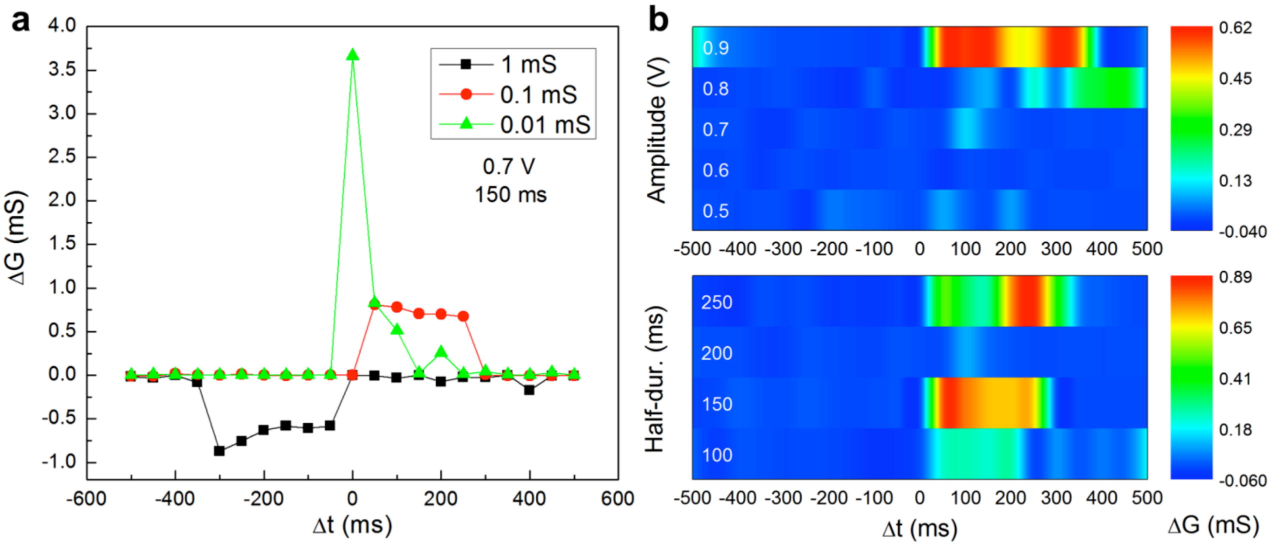

Figure S5. STDP window of Cu/PPX/ITO memristive structures obtained with bi-rectangular pulses. (a) STDP window for various initial conductance values. Post-synaptic pulses were applied after pre-synaptic ones with a varying delay time Δ*t*. All the pulses were symmetric. Every point of the curves is a median of 10 recorded experimental values. Pulse amplitudes and half-durations were 0.7 V and 150 ms, respectively. (b) STDP window for various pulse amplitudes (top image, half-duration is 200 ms) and half-durations (bottom image, amplitude is 0.7 V). Images are obtained by colour mapping of a cubic interpolation (2000 pts) of the corresponding STDP curves (Figures S4–S5).

By analogy, the bi-triangular pulse was checked.

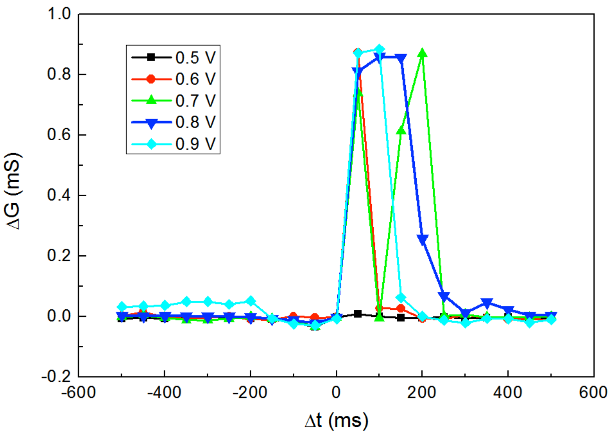

Figure S6. STDP window of Cu/PPX/ITO memristive structures, obtained with bi-triangular pulses of various amplitudes. Post-synaptic pulses were applied after pre-synaptic ones with a varying delay time Δ*t*. All the pulses were symmetric. Every point of the curves is a median of 10 recorded experimental values. Initial conductance was 0.1 mS, pulse half-durations were 200 ms.

Note that the 0.8 V curve is the smoothest and has one extremum, therefore we chose this pulse amplitude for the next experiments.

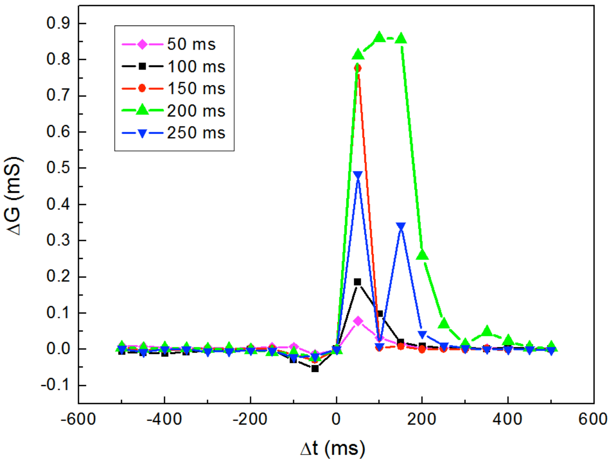

Figure S7. STDP window of Cu/PPX/ITO memristive structures, obtained with bi-triangular pulses of various half-durations. Post-synaptic pulses were applied after pre-synaptic ones with a varying delay time Δ*t*. All the pulses were symmetric. Every point of the curves is a median of 10 recorded experimental values. Initial conductance was 0.1 mS, pulse amplitudes were 0.8 V.

The same experiment parameters (0.8 V, 200 ms) show here the best result in this case.

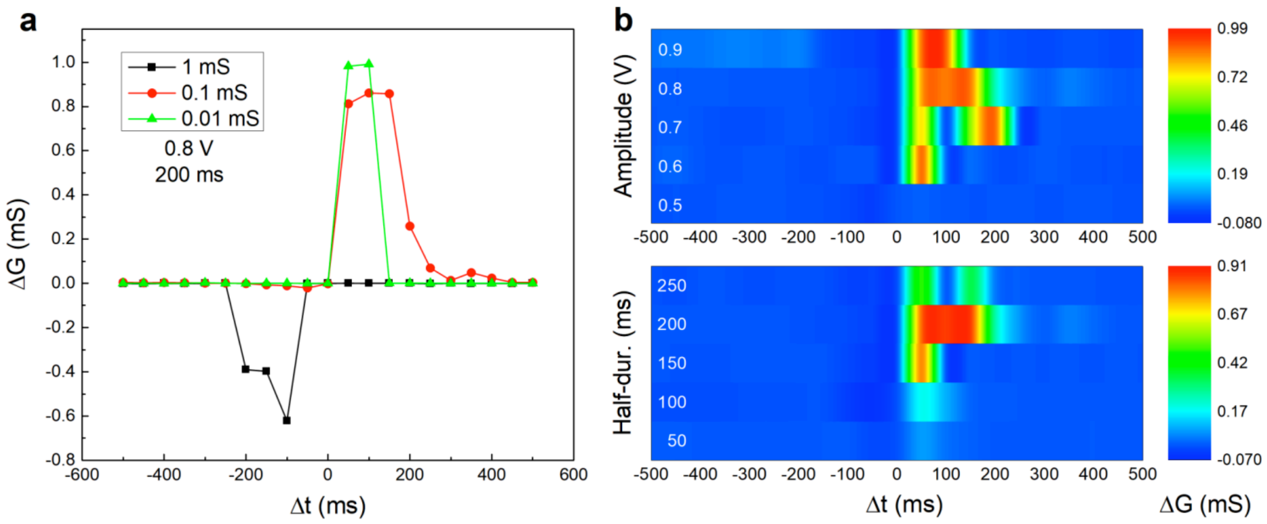

Figure S8. STDP window of Cu/PPX/ITO memristive structures obtained with bi-triangular pulses. (a) STDP window for various initial conductance values. Post-synaptic pulses were applied after pre-synaptic ones with a varying delay time Δt. All the pulses were symmetric. Every point of the curves is a median of 10 recorded experimental values. Pulse amplitudes and half-durations were 0.8 V and 200 ms, respectively. (b) STDP window for various pulse amplitudes (top image, half-duration is 200 ms) and half-durations (bottom image, amplitude is 0.8 V). Images are obtained by colour mapping of a cubic interpolation (2000 pts) of the corresponding STDP curves (Figures S6–S7).
